# Branched Lateral Tail Fiber Organization in T5-Like Bacteriophages DT57C and DT571/2 is Revealed by Genetic and Functional Analysis

**DOI:** 10.3390/v8010026

**Published:** 2016-01-21

**Authors:** Alla K. Golomidova, Eugene E. Kulikov, Nikolai S. Prokhorov, Ricardo С. Guerrero-Ferreira, Yuriy A. Knirel, Elena S. Kostryukova, Karina K. Tarasyan, Andrey V. Letarov

**Affiliations:** 1 Winogradsky Institute of Microbiology, Research Center of Biotechnology of the Russian Academy of Sciences, Leninsky Ave. 33, build. 2, Moscow 119071, Russia; alusik81@mail.ru (A.K.G.); eumenius@gmail.com (E.E.K.); prokhoroff@gmail.com (N.S.P.); tarasyan_k@mail.ru (K.K.T.); 2 Moscow Institute of Physics and Technology, Dolgoprudny, Moscow Region, 141700, Russia; 3 École Polytechnique Fédérale de Lausanne (EPFL), BSP-415, 1015 Lausanne, Switzerland; ricardo.guerrero@epfl.ch; 4 N. D. Zelinsky Institute of Organic Chemistry, Russian Academy of Sciences, Leninsky Ave. 47, Moscow 119991, Russia; yknirel@gmail.com; 5 Federal Research and Clinical Center of Physical-Chemical Medicine of Federal Medical Biological Agency, Pirogovskaya ul., 1a, Moscow 119435, Russia; el-es@yandex.ru; 6 A.N. Severtsov Institute of Ecology and Evolution, Biotechnology of the Russian Academy of Sciences, Leninsky Ave. 33, build. 2, Moscow 119071, Russia

**Keywords:** bacteriophage, T5-like phage, bacteriophage adsorption, phage *in situ* evolution, tail fiber proteins, phage branched adhesin, *E. coli* O-antigen, O-antigen O-acetylation, horse feces

## Abstract

The T5-like siphoviruses DT57C and DT571/2, isolated from horse feces, are very closely related to each other, and most of their structural proteins are also nearly identical to T5 phage. Their LTFs (L-shaped tail fibers), however, are composed of two proteins, LtfA and LtfB, instead of the single Ltf of bacteriophage T5. *In silico* and mutant analysis suggests a possible branched structure of DT57C and DT571/2 LTFs, where the LtfB protein is connected to the phage tail *via* the LtfA protein and with both proteins carrying receptor recognition domains. Such adhesin arrangement has not been previously recognized in siphoviruses. The LtfA proteins of our phages are found to recognize different host O-antigen types: *E. coli* O22-like for DT57C phage and *E. coli* O87 for DT571/2. LtfB proteins are identical in both phages and recognize another host receptor, most probably lipopolysaccharide (LPS) of *E. coli* O81 type. In these two bacteriophages, LTF function is essential to penetrate the shield of the host’s O-antigens. We also demonstrate that LTF-mediated adsorption becomes superfluous when the non-specific cell protection by O-antigen is missing, allowing the phages to bind directly to their common secondary receptor, the outer membrane protein BtuB. The LTF independent adsorption was also demonstrated on an O22-like host mutant missing O-antigen O-acetylation, thus showing the biological value of this O-antigen modification for cell protection against phages.

## 1. Introduction

Bacteriophage T5, infecting many laboratory strains of *E. coli*, is one of the series of seven “authorized” “T” phages selected by Max Delbrück that have served as model organisms for basic research in phage biology since the 1940s [1]. Recently, T5-related viruses were identified also in non-*Enterobacteriaceae* hosts. At the moment, T5-like phages are considered a genus in the *Siphoviridae* family with at least 11 representatives with fully sequenced genomes annotated in GenBank as “T5likeviruses”: coliphages T5, BF23, CEV2, H8, and bv_EcoS_AKF33; *Salmonella* phages SPC35, EPS7, and Stitch; *Yersinia* phage phiR201; *Pectobacterium* phage My1, and *Vibrio* phage pVp1 [2,3,4,5,6,7].

The virion of bacteriophage T5 consists of an isometric icosahedral T = 13 head, containing 105 k.b.p. of genomic DNA, and a long noncontractile tail with a single central tail fiber and three side tail fibers, with the latter also referred to as L-shaped tail fibers (LTF) [8,9]. In other T5-like phages, the morphology of the tail fibers is not always known so one may use the same abbreviation for lateral tail fibers.

T5-like coliphages are widespread in nature, including as components of human and animal intestinal microbiomes [10,11], see also [12,13]. They are also found in downstream habitats such as sewage or soils. While maintained in the gut ecosystem even over a limited period of time, these phages are believed to undergo rapid *in situ* evolution [14]. The analysis of the multiple, closely related isolates recovered from related environmental samples may shed light on the mechanisms of short-term adaptation of the phage genome.

The genome regions that are expected to be a subject to the most intensive selection include the genes coding for host specificity determinants of the bacteriophage [15,16].

The adsorption apparatus of T5 phage has been characterized in substantial detail [17,18,19], see also [9] and references therein. The main T5 receptor is an outer membrane transporter protein, FhuA, receptor-binding protein pb5 or Oad (reviewed in [9]) that is located at the extremity of the T5 straight fiber (pb4) protruding from the conical basal structure at the end of the phage’s long tail [17]. The interaction оf pb5 with FhuA is necessary and sufficient for the triggering of phage DNA ejection [9,20]. Interestingly, gene *llp*, which is found adjacent to the gene *oad*, codes for the receptor-blocking lipoprotein that is expressed in the infected cell and binds to the phage receptor, excluding superinfection and presumably decreasing phage inactivation on cell debris after lysis of the host [21,22].

Phage BF23, which is closely related to T5, has an alternative variant of the host-recognition module with receptor binding protein, Hrs, which is only distantly related to T5 Oad [23]. This protein recognizes another outer membrane transporter, BtuB, that is required for Vitamin B12 uptake [24]. The cognate Llp protein encoded by BF23 genome has no homology with its T5 analogue but performs the same function, blocking BtuB proteins on the infected cell surface, thus preventing superinfection [23].

Phage T5, like other T5-like viruses, carries an additional adsorption device comprised of three L-shaped fibers (LTFs). These fibers are attached to the thin collar at the upper end of the conical basal structure, at the level of the interface between tail tube protein (pb6) and Dit protein pb9 [9,19]. In phage T5, LTFs are formed by pb1 protein [9,17]. It has been demonstrated that LTFs bind specifically to polymannose motif of bacterial lipopolysaccharide (LPS) O-antigen, increasing the rate of the phage adsorption on O-antigen-producing strains [25,26]. The interaction of LTF with LPS is not essential for T5 infection under laboratory conditions and LTF-deficient mutants can grow effectively [26]. However, the rate of adsorption on O-antigen-producing strains is significantly decreased in such mutants while the wild type T5 binds to these cells more actively than to the host lacking the O-antigen polysaccharide [26]. It is unclear whether LTFs contribute significantly to host range determination of the T5-like bacteriophages with respect to coexisting wild bacterial strains in their natural habitats.

Here, we present a characterization of two environmental isolates of T5-like phages. These were recovered from a sample of the horse faeces [14] and, being closely related to each other, have slightly different host ranges [27]. At the same time, these viruses possess the *ltf* loci consisted of two genes instead of one *ltf* gene in bacteriophage T5. The aim of this work was to identify the primary and the secondary (the main) receptors of the phages DT57C and DT571/2, to reveal the functions of two different Ltf proteins encoded in their genomes and to determine the molecular mechanism responsible for the difference of the host ranges of these viruses.

## 2. Materials and Methods

### 2.1. Phage and Bacterial Strains and Their Cultivation

Bacteriophage BF23 was kindly provided by Vladimir Ksenzenko, IBPM, Pushino, Russia. The bacteriophages DT57C, DT57-1/2, and DT530 were isolated from the horse faeces in course of our previous work [14] using *E. coli* C600 as a host and were maintained in our laboratory collection. Standard laboratory *E. coli* strains C600, DH5α, JM109, JM109(DE3), and BL21(DE3) were from our laboratory collection. Environmental *E. coli* isolates 4s, HS½, and HS3-104 were recovered from feces obtained from the same group of horses that served as a source of the abovementioned phages. The animals were stabled together and are believed to freely exchange intestinal phages and bacteria [14]. The species-level identification of these strains was confirmed by MALDI-TOF of whole cells extracts profiling using Biotyper instruments (Bruker-Daltonics, Germany) according to manufacturer’s recommendations.

*E. coli* 4s strain produces an OPS that is similar to the O22 type OPS but has an additional side-chain glucosylation [28]. The mutants of *E. coli* 4s strain, 4sI and 4sR, were selected previously as variants that are resistant to bacteriophage G7C [29,28]. *E. coli* 4sI produces a non-O-acetylated OPS due to mobile element insertion to *wclK* gene while 4SR does not produce O-antigen due to disruption of a synthetic pathway also by a mobile element insertion [28]. *E. coli* HS1/2 strain produces the O87 type OPS, whose structure has been determined [30].

The mutants of the phages used were obtained in the course of this work. Their genotypes and host ranges are given in Table 1.

Bacteria were propagated on LB medium (Trypton 10 g, yeast extract 5 g, NaCl 10 g, water up to 1 L). This medium was supplemented with 15 g of agar per 1 l for plates or with 6 g of agar per 1 L for top agar used for double-layer phage plating.

### 2.2. Bacteriophage Host Range Determination

To determine the ability of phages to grow on particular host strains, 5 µL drops of serial dilutions from 10^0^ to 10^−8^ of phage stock were applied over the fresh double layer plate inoculated by the strain of interest. Before the application of phages, plates were dried open in the laminar hood for 15 min to reduce condensation. Plates were then incubated at 37 °C overnight. The results were registered as follows: (−) no phage growth; (+) growth inhibition zones formed at low dilution with the single plaques visible in the spots of the higher dilution with estimated efficiency of plating (EOP) within 1 order of magnitude compared to the control plate with *E. coli* C600 lawn; (I) growth inhibition zone is visible at lower dilution but it disappeared at higher dilutions without visible plaques formed. If the plaques were formed at EOP lower than 10^−1^, then the approximate value of the EOP was registered.

### 2.3. LPS Analysis

LPS extraction and SDS-PAGE separation and amplification of the O-antigen synthesis cluster were performed as described [28].

### 2.4. Bacteriophage Adsorption

Rates of phage adsorption were determined as described earlier [29]. Briefly, the log-phase cells of the appropriate strain were pelleted by gentle centrifugation at 3000 g for 2 min at room temperature and resuspended in fresh LB medium up to intended OD_600_. Then, phage was mixed to the cell suspension up to the final concentration ca. 2 × 10^3^ PFU·mL^−1^ and the mixture was incubated at 37 °C. At the desired time points, 0.5 mL aliquots were taken and immediately centrifuged on the table top centrifuge at 12,000 g for 1 min. Then, 50–250 μL of supernatant was plated on appropriate host strain lawn for free phage enumeration. The same dilution of the phage stock made in LB medium without the cells was incubated in the same conditions and was plated for estimate the initial PFU count. Alternatively, if the plating was performed on the same strain as used for adsorption, the initial time point could be estimated by plating of an aliquot without centrifugation.

### 2.5. Oligonucleotide Primers and Plasmid Constructions

Primers BF23Llp-R (5′-TGACATATCTTTCATGCTCCT) and BF23Llp42-F (5′-GGAGAGATAAATATGAAAAAGTTTGTAATTGCACTAGTTGC) were used for PCR amplification of *llp* gene. The fragment obtained was cloned into pGEM-T vector (Promega, Madison, WI, USA) according to the manufacturer’s recommendations. The insert orientation was selected to put the *llp* gene under the control of T7 polymerase promoter. This construction was named pLlp-7C.

For introduction of amber mutations into selected genes, long (65–75 nt) mutagenizing primers were designed to provide a ca. 50 nt shoulder for recombination. They were used in PCR together with corresponding conventional primer to generate DNA fragments that were cloned into pGEM-T vector and used for recombination with phages. For mutagenesis of *LtfB* gene of DT571/2 phage, the mutagenizing primers 71/2f1m-F 5′-CGAGTATTTAATTCTTCTTTATCTATAGTGTAGTTAGAGAT**TCACAA**GTATTTAGAAGTTAGTGCAACAG (the altered residues are shown in bold) and 71/2f1m-R (5′-AGCACTACTTCTGACGAGCG) were used, resulting in Q45*opal* mutation and change of the adjacent Ile codon to an alternative one to facilitate PCR detection. Primer 71/2f1mInd-F (5′-GTGTAGTTAGAGATTCACAA) in combination with 71/2f1m-R was used for PCR screening of the progeny phage plaques for the recombinants.

Similarly the primers 71/2f2m-R (5′-ATTCTGAACATAGATGACACTACAACTACTGCT**TAG** AAGTATCCTAAATATACAGTAG-3′), 71/2f2m-F (5′-GATGCTGCTCAAGGTGCTG) were used to introduce S23am mutation into LtfA gene of DT571/2 phage and the primers 71/2f2mInd-R (5′-CAC TACAACTACTGCTTAG) and 71/2f1mInd-F (5′-GTGTAGTTAGAGATTCACAA) served for the PCR screening.

Primers T5f2-F (5′-GTTAGTGCAACAGCACCAGC) and T5f2-R (5′-GATATTGCTACCACGTATAC) were used for amplification of the divergent regions in LtfA genes in DT571/2 and DT571/2 with the flanking conserved sequences. The divergent regions in LtfB gene of these phages were amplified using T5f1-F (5′-CCTGTGGTCTTACAACGTCC) and T5f1-R (5′-TGCCTAAATCCGGCGCAATG) primers. The PCR fragments were cloned and used for recombination with target phages to switch their host ranges (see Results section). Primers BtuB-F (5′-CCAACGTCGCATCTGGTTC), BtuB-F2 (5′-GTAACGCTGTTGGGCGAT) and BtuB-R (5′-GATCTCGTCATAGACCGA) were used for amplification and sequencing of btuB genes from the bacterial strains.

### 2.6. Targeted Phage Mutagenesis Using Recombination with the Plasmids

To introduce plasmid-encoded mutations into the phage genomes, we used high copy number vectors based on pGEM-T. The appropriate plasmid was electroporated into *E. coli* C600 or *E. coli* JM109 cells, with liquid culture of the transformants grown and infected by the appropriate phage at MOI of 0.01–1.0. The culture was incubated at 37 °C with vigorous agitation until visible lysis had occurred. A drop of chloroform was added and the lysate was plated on *E. coli* C600 lawn. To detect recombinant phages, plaques were transferred by toothpicks to plates pre-inoculated with a fresh lawn of test cultures. Loss of growth was expected for the mutants, while the plate with *E. coli* C600 lawn, expected to be permissive for all the mutants generated in this work, was used as a control. Alternatively, for the recombinants expected to gain the ability to form plaques on strains restrictive for the parental phage, the lysate obtained after recombination was plated directly on such strain. In the case of *ltfB* mutants, the phenotype of which could not be anticipated, the plaques from the primary plating were transferred onto a new C600 plate to obtain larger plaques. These plaques were cut out of the top agar using glass capillaries of ca. 0.7 mm diameter. Phages were eluted in 300 µL of physiological saline over 40 min at room temperature. The resulting extracts were then centrifuged using a tabletop centrifuge at maximum speed for 3 min and supernatants were used for PCR screening with one of the primers designed to hybridize by its 3’ end to the altered nucleotide positions. The identified mutant phages were purified by a single plaque re-isolation and confirmed by the targeted sequencing of the mutated locus.

### 2.7. Selection of Phage-Resistant Bacterial Clones

Selection for bacterial mutants that are resistant to specific phage types was performed using conventional double-layer plating with appropriate phage lysate containing approximately 10^9^ PFU added in top agar instead of bacterial culture. Three microliters drops of bacterial culture containing 10^6^–10^7^ CFU were spread over the surface of the phage-containing agar with a glass spatula and the plates were incubated overnight at 37 °C. Resulting colonies were streaked out to the fresh LB plates and then purified by repeated single colony isolation. Absence of associated phage was confirmed by streaking the obtained resistant cultures over the lawn of the original phage sensitive strain.

### 2.8. Phage Morphology Visualization

For negative staining electron microscopy, phage samples were absorbed onto a glow-discharged, 400-mesh, carbon-coated copper grid. After 1 min, excess sample was blotted away and remaining phages were stained with 1% uranyl acetate water solution for 1 min. Micrographs were acquired on a FEI Tecnai F20 electron microscope (FEI, Hillsboro, OR, USA) operated at 200 kV with magnifications ranging from 50,000× to 100,000×. Images were collected using a 4 k × 4 k FEI Eagle CCD camera. Transmission electron microscope magnification was calibrated using a crossed-line grating replica (Electron Microscopy Sciences. Hatfield, PA, USA).

For cryo-electron microscopy, four 4 μL aliquots were applied onto glow-discharged Quantifoil grids with hole size of 2 μm (Quantifoil Micro Tools, Großlobichau, Germany) and subsequently blotted for 3 s before plunging into liquid ethane using an FEI Vitrobot Mark IV (FEI, Hillsboro, OR, USA). Images were collected on a FEI Tecnai F20 (FEI, Hillsboro, OR, USA) operated at 200 kV and using a 4 k × 4 k FEI Eagle CCD camera at a total electron dose of 20 electrons/Å^2^ and a range of defocus between −2 and −4 μm. Micrographs were acquired at magnifications ranging from 50,000× to 100,000×.

### 2.9. Accession Numbers

The nucleotide sequence of phage DT530(1) ltf locus is deposited to GenBank under the accession number KU236381 the *ltf* locus of phage DT571/2-ABF under the accession number KU159289, the sequences of the mutated btuB genes from two *E. coli* 4sR ltfAR strains are deposited under the accession numbers KU291214 and KU291215.

## 3. Results

### 3.1. Bacteriophage Host Ranges

In the course of our ecological project performed in 2006, a series of five bacteriophages closely related to coliphage T5 were isolated from a single sample of the horse feces on lawns of the laboratory *E. coli* C600 strain [14]. Random clones of these phage’s DNA shared more than 95% identity at the nucleotide level with T5 phage and also possessed similar but not identical genomic DNA restriction profiles [14].

Later, one of these isolates, the phage T5-C7, was recognized to be a mixture of two highly related phages that were originally isolated from a single plaque and remained associated despite multiple passages performed in the course of our work with this isolate. Extensive restriction fragment length polymorphism (RFLP) analysis proved that these viruses are distinct isolates that could not have evolved from a single phage genotype as a result of one or few mutation(s). One of the phages was then named DT57C and the other DT571/2.

The host ranges of these bacteriophages are very similar (Table 1) with the exception of their ability to infect two environmental *E. coli* isolates obtained from the same animal [14]. Phage DT57C forms plaques on *E. coli* 4s that was previously described as an unique host for N4-like bacteriophage G7C [29] but not on the strain HS1/2. Phage DT571/2 host range is the opposite—it grows on *E. coli* HS1/2 but not on *E. coli* 4s strain. Both phages were able to grow on derivatives of *E. coli* 4s, the strains 4sI and 4sR which were selected for resistance to bacteriophage G7C and which are deficient for O-antigen O-acetylation or O-antigen biosynthesis, respectively [28]. Both strains also were able to grow on the environmental isolate HS3-104. The phages also are infective for all laboratory strains tested (C600, NM522, Be/1, BL21(DE3), JM109 and JM109(DE3)).

**Table 1:** Efficiency of plating (EOP) of bacteriophages and their mutants on different *E. coli* host strains. The strain 4sR ltfA^R^ is a mutant of *E. coli* 4sR selected for resistance for DT571/2 *ltfA* *am* phage mutant (two independent clones having different mutations in *btuB* gene had the same phenotype so this strain designation stays for both of them).

Bacteriophage Strains	Bacterial Host Strains							
	4s	4sR	4s*ltfA*^R^ ltfA^R^	4sI	4sI:pwclK	HS1/2	C-600	3/104
BF23	−	+	−	+	−	−	+	−
DT571/2	−	+	−	+	−	+	+	+
DT5712 *ltfA am*	−	+	−	+	−	−	+	−
DT5712 *ltfB opal*	−	+	−	+	−	10^−1^^b^	+	10^−5^^a^
DT571/2-ABF	−	+	−	+	−	−	+	+
DT57C	+	+	−	+	+	−	+	+
DT57C(4s-)	I	+	−	+	I	−	+	I
DT530	+	+	−	+	+	−	+	+
DT530(1)	−	+	−	+	−	−	+	I

“+“—Efficiency of plating (EOP) >0.5; “−“—No phage growth visible in the spot; “I”—No plaques formed, but the growth inhibition zone appears if a drop of the concentrated phage stock is applied onto the lawn; ^a^ EOP = 10^−5^, normal plaques formed; ^b^ In fresh lysate EOP of ca. 10^−1^ was observed that dropped after the phage purification. Plaques are very small and turbid.

The bacteriophage DT530 has a slightly different restriction profile [14], but its host range is identical to that of the phage DT57C (Table 1). Its derivative, DT530(1), which was spontaneously selected in the course of propagation in the laboratory (see below), has a narrower host range being unable to infect efficiently HS3-104 host that is sensitive to both DT57C and DT571/2 phages (Table 1). To ensure that the resistance of *E. coli* 4s and *E. coli* HS1/2 to phages DT57-1/2 and DT57C, respectively, is due to phage inability to adsorb into these cells and is not a result of the intracellular phage development inhibition, we performed the adsorption experiment for each of two phages on the *E. coli* strains C600, 4s, 4sI, 4sR, HS1/2 and HS3-104. Both DT57C and DT571/2 phages adsorbed efficiently on C600, 4sI, 4sR and HS3-104. DT57C adsorbed on *E. coli* 4s but remained unassociated with the cells if incubated with HS1/2 strain, while DT571/2 behaved inversely adsorbing efficiently on HS1/2 but not on 4s host strain cells. These results indicate that the host range of our phages on the host strain set used is determined by their specificity of adsorption.

### 3.2. Genetic and Functional Analysis of the Adsorption Apparatus

The whole genomes of the bacteriophages DT57C and DT571/2 were sequenced and annotated [27] and the genes of proteins potentially involved in the host recognition were identified.

#### 3.2.1. Host Recognition Protein and Receptor Blocking Lipoprotein

**Figure 1:**
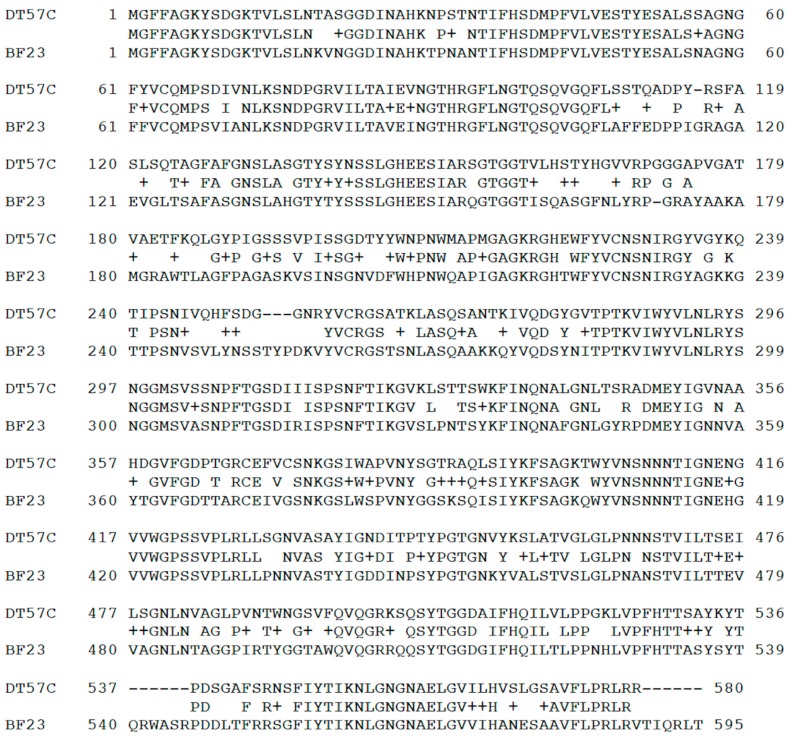
Alignment of Hrs protein (gp DT57C_000128) sequences of phage DT57C and phage BF23 Hrs (DT571/2 Hrs protein is identical to that of DT57C phage).

All phages of our T5-like series possess the same gene sequence for the main receptor binding protein pb5 (Hrs), which is closer to that of phage BF23 than of phage T5. No a.a. polymorphisms were observed between our isolates. In contrast to the bulk of the genome that is highly similar to T5 at the nucleotide level, *hrs* gene nucleotide sequences (that are identical in DT57C and DT571/2 phages) are quite divergent from both T5 and BF23 homologs. The amino acid sequence identity with the phage BF23 counterpart is mosaic (Figure 1) with large divergent patches in the first N-terminal 200 amino acid residues (a.a.) that are believed to be involved in receptor recognition [31,32]. At the same time, the adjacent *llp* genes (that are also identical in our phages) coding for the receptor blocking lipoprotein are almost identical to gene *llp* of phage BF23 at both nucleotide and protein levels (a single a.a. substitution is present).

The decreased a.a. and nucleotide sequence similarities of *hrs* gene (with respect to the genomic neighbourhood) between our phages and phage BF23 suggests that this version of the *hrs* was a relatively recent acquisition by the BF23-like ancestor of our phages (or *vice versa*). The putative recombination point is located between *hrs* and *llp* genes, within the functional module defined by Mondigler *et al.* [23]. To test if the functional fit of the Hrs protein and Llp proteins is conserved, we cloned the *llp* gene of DT57C phage into the pGEM-T vector under the control of T7 promoter. The phage inhibiting activity of Llp expression was measured without IPTG induction limiting the *llp* expression to the level of the promoter leakage. BL21(DE3) strain was used for comparison of our phages with phage BF23, and the *E. coli* JM109(DE3) strain was used for comparison with phage T5 since *E. coli* BL21(DE3) was found to be resistant to T5 infection. The expression of DT57C *llp* inhibited infection of the cells by all our isolates and by phage BF23, reducing the efficiency of plating (EOP) more than 100-fold.

It has to be noted that under the conditions employed, the effect of *llp* gene expression against our phage isolates as well as against BF23 phage was less pronounced than as reported by Mondigler *et al.* [23]. In their experiments, a decrease in EOP of more than six orders of magnitude was observed compared to only two orders of magnitude under the conditions employed in this study. The reasons for this difference are not clear.

Bacteriophage T5 infection was not affected by the phage DT57C gene *llp* expression at all. These results indicate that BtuB blocking protein Llp of BF23-type binds also to the receptor of our phages. We so suggested that Hrs protein in our isolates recognizes the same receptor as BF23 phage Hrs protein (BtuB), despite substantial differences in their a.a. sequence. The Llp protein should remain functional for superinfection exclusion in this context. This explains why the progeny of recombination within the functional module was not eliminated by natural selection. In agreement with this conclusion, mutants of *E. coli* C600 selected for BF23 resistance were shown to be resistant to our phages but not to phage T5 (Vladimir Ksenzenko, personal communication) and mutants of the *E. coli* 4sR strain selected for resistance to DT571/2 *ltfA^−^* mutant were resistant to BF23 phage (see below; Table 1). We sequenced *btuB* gene of *E. coli* 4s, *E. coli* HS½ and *E. coli* HS3-104 strains. The inferred a.a. sequences were identical and non-distinguishable from this gene in the majority of published *E. coli* genomes, including the type strain *E. coli* MG1665 sequence. We selected clones of *E. coli* 4sR for the resistance of *ltfA am* mutant completely lacking LTF function (see below) that is thus believed to infect the host cells only via the direct recognition of the secondary receptor by its Hrs protein. All clones tested (*n* = 24) gained simultaneously the resistance to phage BF23 infection. Since we observed no phenotype differences between these clones, we designate all of them 4sR *ltfA*^R^ . Sequencing of *btuB* gene in two randomly chosen 4sR *ltfA*^R^ clones revealed a large deletion in one case and the IS mobile element insertion in another case, both inactivating the gene. Taking all the data together, we conclude that DT57C and DT571/2 utilize BtuB as the secondary receptor. At the same time, this receptor recognition cannot be responsible for the host range differences between DT57C and DT571/2 phages.

#### 3.2.2. Lateral Tail Fibers Locus

Phages DT57C and DT571/2 both have two large genes coding for the tail fibers *versus* one such gene (g 143 or *ltf*) in phages T5 or BF23. These genes were named *ltfA* and *ltfB* according to their positions with respect to the transcription order [27]. Analysis of the published genome annotations and blastP search revealed that double fiber genes arrangement is shared by several other T5-related bacteriophages—enterobacteria phages phiR201, SPC35, and vb_EcoS_AKFV33 carry two *ltf* genes while phages T5, BF23, H8 and EPS7 possess only one fiber protein, related through its N-terminal part to LtfA of our phages.

**Figure 2:**
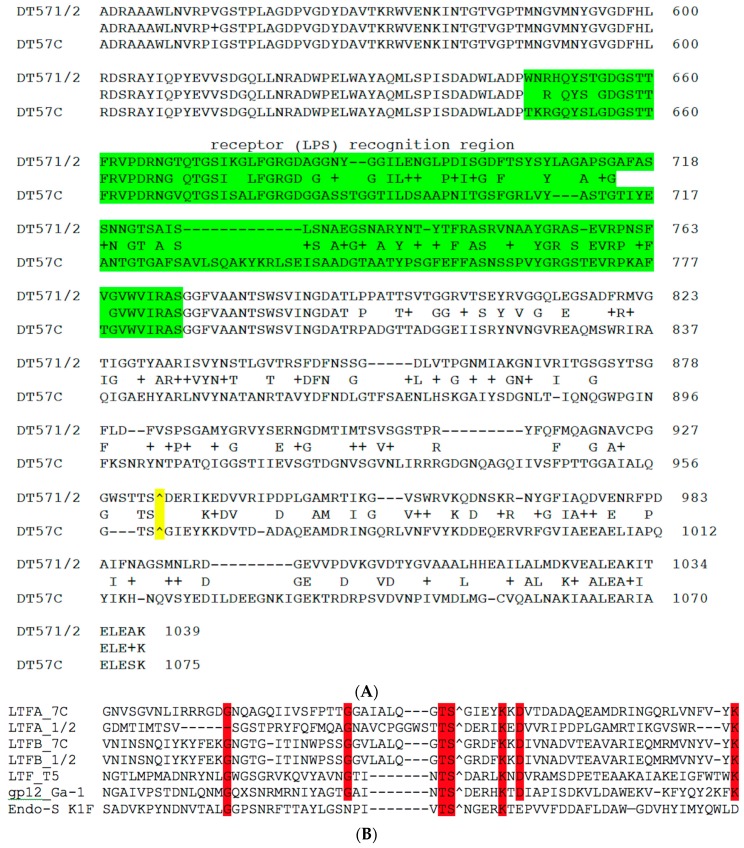
(**A**) BlastP alignment of the C-terminal fragments of LtfA proteins from phages DT57-1/2 and DT57C. The fragment that was transmitted by recombination with plasmids (see explanations in the text) to switch host specificity is highlighted in green. The predicted autocleavage site is marked by “^” and highlighted in yellow; (**B**) ClustalV alignment of the regions neighboring the putative cleavage site of the LtfA and LtfB proteins of our phages and in proteins with known structures of the autocleavable C-terminal chaperone domain: Ltf of bacteriophage T5 (X69460), gp12 of Bacillus subtilis bacteriophage Ga-1 (NC_002649) and endo-sialidase of *E. coli* bacteriophage K1F (AJ505988). “^” indicates the predicted or experimentally determined autocleavage sites. Red highlight indicates the residues conserved in six or more out of seven aligned sequences.

The *ltfB* genes are almost identical in DT57C and DT571/2 phages except for a short stretch of divergence in the N-terminal part, between 65 and 90 a.a. residues (DT57C coordinates). The N-terminal halves of the *ltfA* genes are also highly related in our phages. Their very N-terminal 80 a.a. domains share 63% of identity with the corresponding domain of the Ltf protein of bacteriophage T5 mediating the fiber attachment to the T5 virion’s tail. At the same time, LtfA proteins of our phages have a long region of a.a. sequence divergence at the C-terminal parts of encoded proteins (Figure 2A). We suggest that this protein region may be involved in the host recognition.

**Figure 3:**
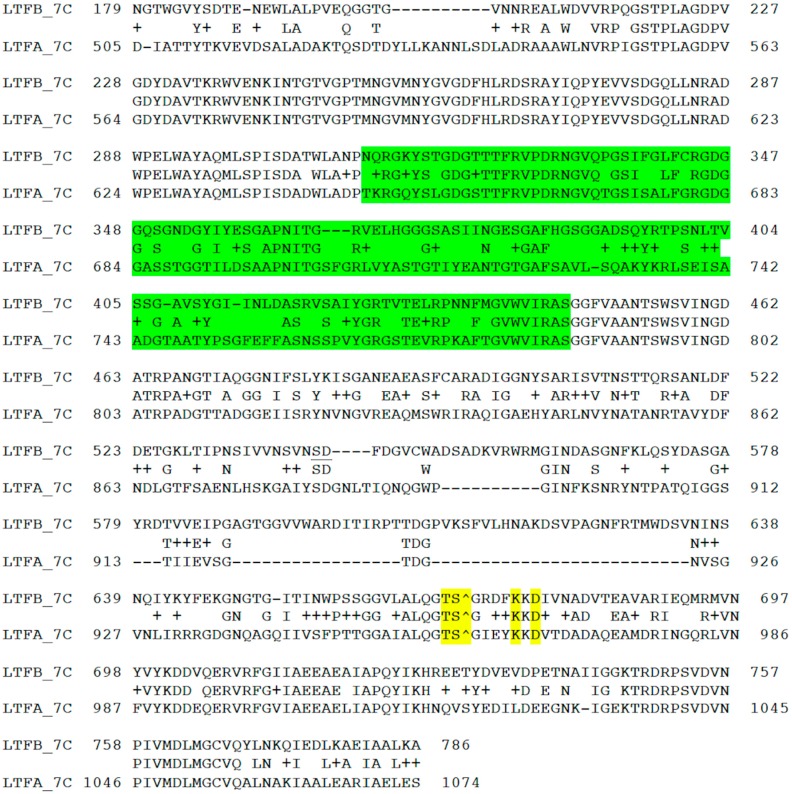
Alignment of the C-terminal regions of LtfA and LtfB proteins of the phage DT57C. The green highlight indicates the region that determines the receptor binding specificity (experimental evidence for LtfA only). The predicted autocleavage site is marked by “^”. The conserved residues near the cleavage site are highlighted in yellow.

Despite the observed sequence divergence between LtfA and LtfB proteins and between C-terminal moieties of LtfA proteins of two phages (Figure 2 and Figure 3), all the above-mentioned proteins carry on their C-terminal extremities the predicted conserved protease domains, pfam13884, that are related to bacteriophage receptor recognition proteins’ C-terminal chaperone domains that are autocleavable by their own peptidase activity. The cleavage sites can be easily predicted on the basis of the conserved motif present in all proteins [33]. A similar autocleavable chaperone domain was found also at the C-end of bacteriophage T5 Ltf protein [33] (Figure 2B).

**Figure 4:**
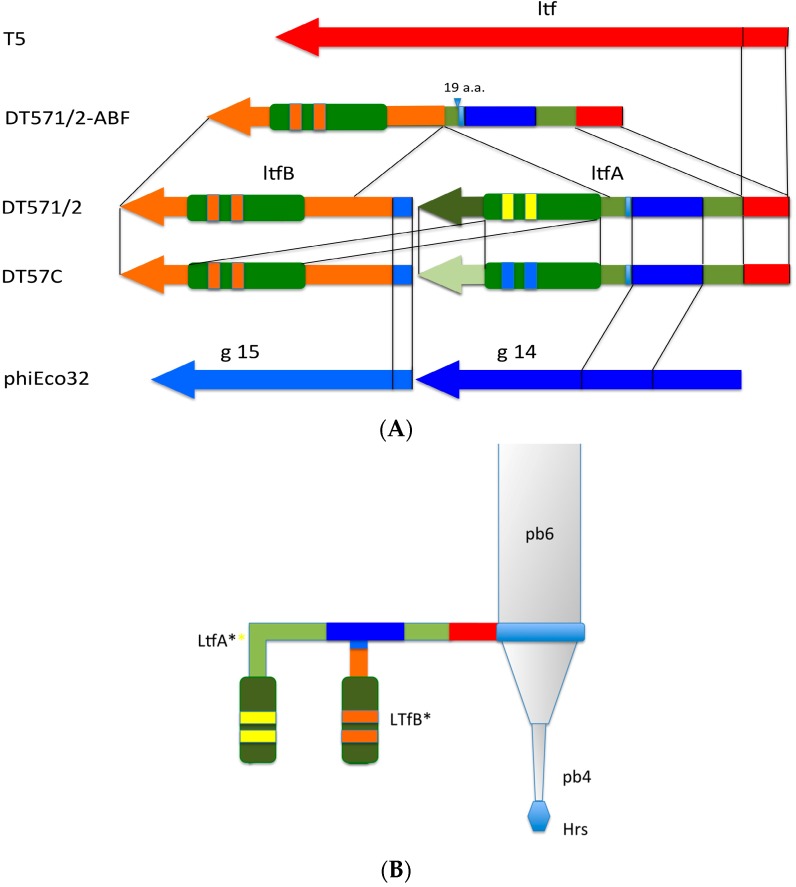
(**A**) Organization of the tail fiber loci in bacteriophages T5, DT57C, DT57-1/2, DT571/2-ABF, and (partially) phiEco32. The similarity of the a.a. sequence regions in LTFs of T5-like phages with other viruses are indicated by similar colors. The green rectangles with double bars indicate the receptor binding regions in Ltf proteins, the colors of these double bars indicate one of the three types of the Ltf receptor recognition domains: yellow LtfA-DT571/2, blue LtfA-DT57C, orange LtfB specific for O81-like O22-like and to O87-like OPS, respectively. Narrow light blue band indicates the block of tandemly repeated 19 a.a. motifs in LtfA protein; (**B**) Proposed model of the branched LTF organization. Only one of three LTFs is shown. The asterisk indicates that corresponding protein is proteolitically processed. The thick rectangles with double bars indicate the receptor binding regions in Ltf proteins. Dark blue and light blue parts stay for the regions of homology with phiEco32 phage gp 14 and gp 15, respectively, red part—the region of homology to phage T5 Ltf.

The middle and C-terminal regions of the LtfA and LtfB protein sequences from our phages share homology with a number of bacteriophage encoded fiber-proteins. It has to be specifically mentioned that the region between a.a. positions 223 and 471 in LtfA that is conserved between phages DT57C and DT571/2 is related to the similarly positioned (a.a. 239–508) region of phiEco32 gp 14 tail fiber protein. At the same time, the very N-terminal 45 a.a. of the LtfB protein are highly similar (32 out of 45 residues are identical) to another phiEco32 tail fiber protein gp15 (Figure 4). As in phiEco32 phage the proteins gp14 and gp15 interact to form a branched fiber (Cryo-EM reconstruction data; Peter Leiman, EFPL, Lausanne, Switzerland, personal communication), one can suggest that LtfA and LtfB proteins of our phages do also interact. In this case, the LtfA protein would mediate attachment of LtfB to a viral particle, thereby explaining how LtfB completely lacking any sequence homology to T5 proteins interacts with the virion tail formed by the proteins virtually identical by their a.a. sequences to bacteriophage T5 tail components.

### 3.3. Host Range can be Modified by Recombination within ltfA Gene

The divergent regions of *ltfA* and *ltfB* from DT57C and DT571/2 phages were cloned with the flanking sequences that are identical in these phages to generate the plasmids pltfA-7С, pltfA-71/2, pltfB-7C, and pltfB-71/2. *E. coli* C600 cells were transformed by these plasmids and the transformants were used as hosts to produce of DT57C and DT571/2 phage stocks. The phage progeny originating from recombination with the plasmids that was expected to have switched host specificity was detected by plating on heterologous hosts (i.e., *E. coli* 4s for the phage DT571/2 and *E. coli* HS½ for the phage DT57C). Such recombinants were detected with the plasmids pltfA-7C (for DT571/2 phage) and pltfA-71/2 (for DT57C phage) at the frequencies of about 10^−4^.

The recombinants were purified by repeated single plaque isolation and tested on both host strains mentioned above. It was shown that they lost the ability to form plaques on the strains permissible for the parental phages but gained the ability to infect the host of the phage-donor of the *ltfA* sequence.

Re-sequencing of the *ltfA* genes from two recombinant phage clones revealed that only a part of the divergent nucleotide sequence had been acquired, indicating that the determinant of host specificity is narrower than the whole of the divergent C-terminal region (Figure 2). Interestingly, the sequence of the identified receptor recognition site of the LtfA protein of DT57C phage matches at 82% identity the a.a. sequence of the fiber protein from podovirus phiKT, previously isolated by us from the same horse (our unpublished data; see GenBank NC_019520.1 for the genome sequence). This observation is in good agreement with phage phiKT ability to grow on *E. coli* 4s cells [28].

### 3.4. LtfA Mutants

We characterized an occasionally isolated spontaneous mutant of DT57C phage that was unable to form plaques on the *E. coli* 4s strain but produced growth inhibition in spot-test if concentrated lysate is applied onto the lawn (Table 1). The same behaviour was observed with *E. coli* strain HS3-104 that is sensitive to both DT57C and DT571/2 phages. The sequencing of the phage mutant genome revealed a 3 bp in-frame deletion that led to a deletion of A198 a.a. residue in the LtfA protein. The recombination with the plasmid containing the wild type sequence of this region restored the phage’s ability to adsorb on the *E. coli* 4s host.

We also attempted to generate a mutant phage DT571/2 that is deficient in LtfA protein production, again by recombination with a plasmid containing a mutated sequence. The vast majority of the phages that had the expected phenotype (lost the ability to grow on *E. coli* HS1/2 host) were, however, PCR–negative for the expected mutation. In a control experiment, we picked at random several hundred plaques of DT571/2 phage grown on *E. coli* C600 lawn onto HS1/2 plate and observed that about 30% of the clones had spontaneously lost the ability to infect this host. We thus presumed that LTF-less mutants arise at an unexpectedly high rate in this phage strains and/or they presumably have a significant selective advantage if grown on *E. coli* C600.

Performing the phage-construct plasmid-recombination method with the HS1/2 strain, with subsequent plating of the progeny onto *E. coli* C600 lawn, we were able to reduce the background level of the spontaneous mutations and therefore select the desired *ltfA* S23*am* - containing mutant. This mutant was unable to grow on *E. coli* HS1/2 and *E. coli* HS3/104 but retained the ability to form plaques on *E. coli* C600 and *E. coli* 4s mutants lacking O-antigen production or deficient for O-antigen O-acetylation (Table 1). It appears then that the presence of LtfA protein is essential for both HS1/2 and HS3-104 strain infection. In contrast to the growth on HS1/2 host strain, the type of the LtfA receptor binding domain is not important on HS3/104 host.

### 3.5. The Function of LtfB

The phage infection of the cells containing pltfB plasmids (see above) yielded no recombinant phages featuring switched or expanded host range, indicating that the divergent part of the LtfB protein is not involved in host recognition as was expected from its N-terminal location. To reveal the function of LtfB protein we generated a LtfB-lacking mutant of DT571/2 phage. Since the phenotype of this mutant could not be predicted we used PCR to screen the phage plaques for recombinants. The generated *ltfB* Q45 *opal* mutant was identified, plaque-purified, confirmed by target sequencing, and the host range of this mutant was tested (Table 1). This mutant lost the ability to grow on *E. coli* HS3-104 strain (the observed EOP of 10^−5^ was due to revertants) but formed small turbid plaques on an *E. coli* HS1/2 lawn at the EOP of ca. 10^−1^ that dropped, however, 100–1000 times if the phage was CsCl-purified. The re-sequencing of the *ltfA* gene revealed no alterations. The phages extracted from the plaques formed by *ltfB* *opal* mutant on *E. coli* HS1/2 lawn retained a similar phenotype suggesting that they were neither revertants nor secondary mutants selected on this host. Moreover, stocks of the mutant produced on *E. coli* HS1/2 host showed decreased EOP on this strain with respect to *E. coli* C600.

**Figure 5:**
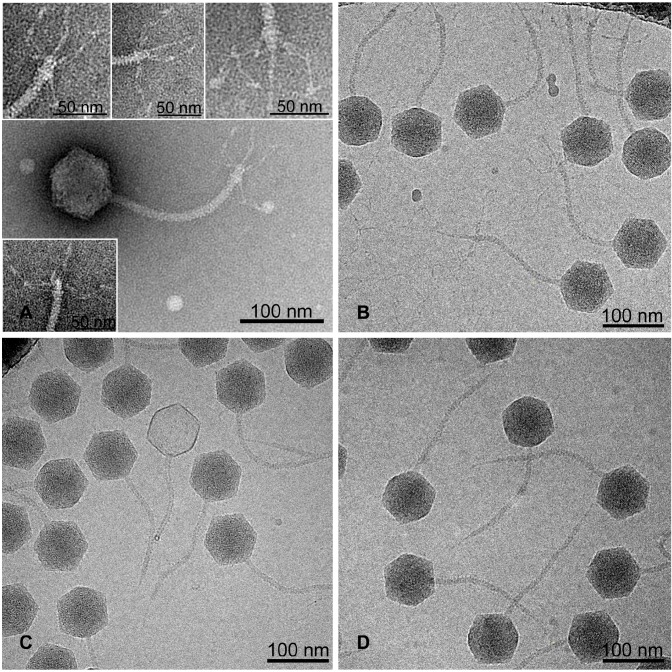
The morphology of DT571/2 phage and its *ltf* mutants. **A**. wild type phage (wt) negative staining; **B**. wt cryo-EM; **C.** *ltfA^−^* cryo-EM; **D.** *ltfB^−^* cryo-EM. The morphology of the wt LTFs appears more structurally complex than “simple” L-shaped fibers of bacteriophage T5 [17].

The inactivation of the *ltfB* gene in DT571/2 thus leads to a decreased viability of the phage (Table 1) if it is plated on O-antigen producing strain HS1/2, and its growth is completely abolished on the strain HS3-104. At the same time, the LtfA deficient mutant is completely unable to grow on both hosts. *LtfB* inactivation did not alter the phage’s growth neither on the laboratory strains (see Materials and Methods) nor on *E. coli* 4sR and 4sI mutants, suggesting that lateral tail fiber function is dispensable on these strains. Electron microscopy of the *opal ltfB* mutant revealed the absence of any LTFs (Figure 5). The morphology of the *opal* ltfB mutant was indistinguishable from the virion morphology in *am ltfA*.

These features of *am* *ltfA* and *opal* *ltfB* mutants, taken together with the fact that this type of LtfA host recognition domain (DT57C-like or DT571/2 like) is not important for the growth on *E. coli* HS3-104, supports our hypothesis that LtfB protein is responsible for recognition of *E. coli* HS3-104 cells and is attached to the virion via LtfA protein. At the same time, one can conclude that the receptor recognition by LtfB is dispensable for the infection of the O-polysaccharide (OPS) protected host *E. coli* HS1/2 by the phage DT571/2 and similarly of *E. coli* 4s by the phage DT57C.

### 3.6. Analysis of E. coli HS3-104 LPS

**Figure 6:**
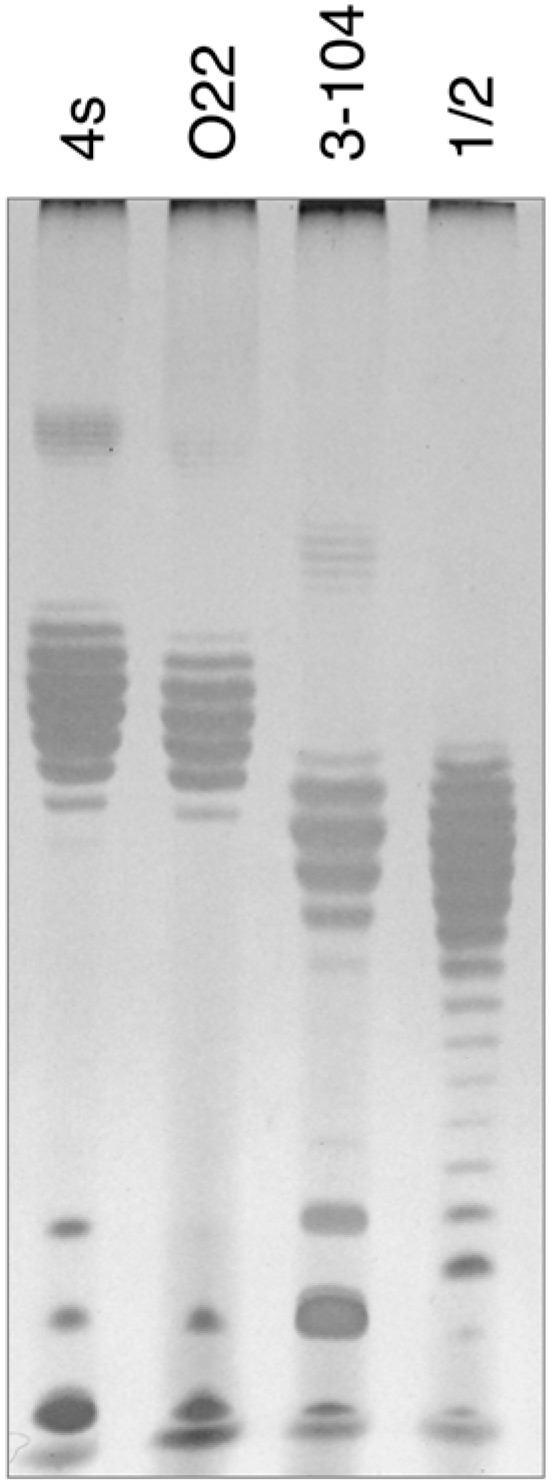
The SDS-PAGE analysis of LPS extracted from *E. coli* strains 4s (O22-like), O22, HS3-104 and HS1/2 (O81 or O81-like). It is clearly seen that the O-unit motility of HS3-104 O-antigen is distinct from all the other strains (the distances between the neighbour bands that correspond to the LPS molecules that differ by a single O-unit are slightly different that leads to the increasing phase shift between the patterns in different lanes on the gel).

In order to elucidate the probable nature of LtfB receptor, we purified LPS from *E. coli* HS3-104 cells and compared it by SDS-PAGE to LPS preparations from *E. coli* strains 4s, 4sR, 4sI and HS1/2 (Figure 6). The results indicate that *E. coli* HS3-104 produces OPS and the pattern formed by the strain 3–104 LPS is significantly different from the other strains suggesting that the O antigen type of this strain is different from that of 4s or HS1/2 strains. We amplified OPS synthesis gene cluster by PCR as described earlier [30] and partially sequenced it from the same primers. The NCBI BLASTn search revealed that the sequences obtained closely match the *E. coli* O81 OPS synthesis gene cluster. Therefore, the O81 (or O81-like) type OPS is likely to be a receptor molecule recognized by LtfB protein.

### 3.7. Purified LPSs do not Inactivate Phages DT57C and DT571/2

The incubation of LPS preparations from *E. coli* 4s and HS1/2 strains with phage particles did not lead to any decrease in bacteriophage titer. No degradation of O-antigen chains was detectable by SDS-PAGE, which indicates that the fibers have no OPS hydrolase enzymatic activity. These results are in agreement with our conclusion that LPS represents primary receptors for phages DT57C and DT571/2 but does not trigger directly DNA release from the virions.

**Figure 7:**
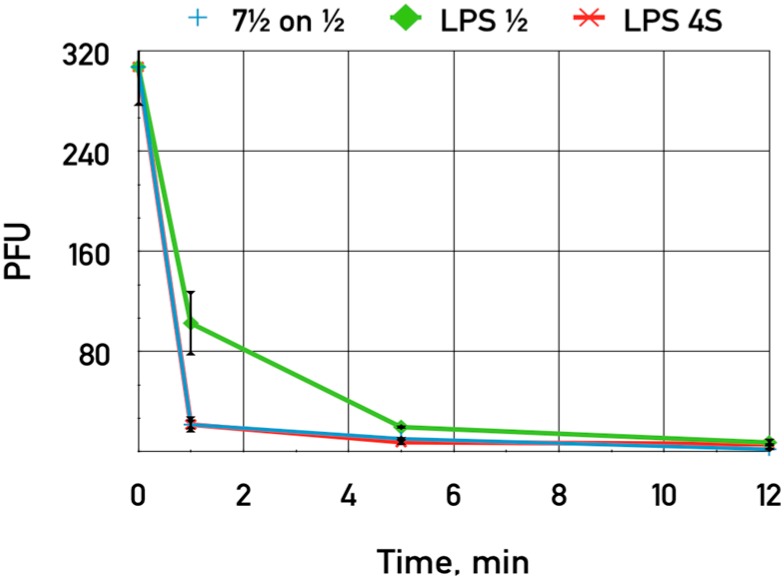
The influence of LPS preparations on the adsorption of bacteriophage DT571/2 on *E. coli* HS1/2 cells. Blue crosses—control without LPS added, green diamonds—LPS extracted from *E. coli* HS1/2 added, red stars—LPS from *E. coli* 4s added. The error bars indicate the SD values.

The purified LPS of *E. coli* HS1/2 adding up to 0.06 mg·mL^−1^ was shown to slightly inhibit the adsorption of phage DT571/2 on these hosts (Figure 7). This LPS preparation did not influence phage DT571/2 adsorption on *E. coli* 4sR cells. Heterologous LPS exerted no influence on phages DT571/2 adsorption. We did not observe any inhibition of phage DT57C adsorption on *E. coli* 4s by any of the LPS preparations. Purified LPS from *E. coli* HS3-104 also did not influence significantly the adsorption of our phages on this strain. These findings indicate that the affinity of LTFs to their receptors in the purified form is relatively low.

### 3.8. Natural Genetic Variability of the ltf Locus

The C-terminal regions of *ltfA* and *ltfB* genes of our phages are related at the level of a.a. and nucleotide sequences. This likeness is more pronounced in DT57C phage (Figure 3). Large patches of divergence are also present, which could explain the differences in receptors’ recognition by these proteins. Nevertheless, the existing long direct nucleotide repeats in these two genes may suggest that the *ltf* locus should be a potential mutation hot spot due to frequent recombination events between these repeats. This instability should be especially high in DT57C where the level of identity between two genes is higher.

We determined the *ltf* locus sequence of phage DT530 that belongs to the same series of environmental isolates as DT57C and DT57-1/2. This phage was cultivated in our laboratory on *E. coli* C600 through several passages since its isolation in 2006. The sequence of its *ltf* gene is almost identical to the *ltf* locus sequence of DT57C phage but contains a large in-register deletion that joined the *ltfA* and *ltfB* genes into a single ORF. Apparently, this deletion was generated as a result of homologous recombination between identical sequences in the genes *ltfA* and *ltfB* and was then selected due to prolonged cultivation on *E. coli* C600 strain. We re-purified phage DT530 from the original stock and re-sequenced its *ltf* locus. The sequence was very close to the phage DT57C *ltf* locus and contained two separate *ltf*A and *ltfB* genes. The originally sequenced deletional mutant of the phage DT530 was named DT530(1), and the name DT530 was assigned to the parental strain containing intact lateral fiber genes.

In bacteriophage DT530(1), the deletion apparently lead to impaired LTF folding and/or assembly or yielded a structurally invalid fiber. This can explain the weak growth of this virus on *E. coli* HS3-104, and this phage was originally picked for analysis due to this phenotype (Table 1).

To search for naturally occurring deletions that would join LtfA and LtfB proteins but would result in more active fused LTF protein, we transferred by toothpicks approximately 200 plaques of DT571/2 *opal ltfB* phage grown on *E. coli* C600 that is permissive host for all the *ltf* mutants to the lawns of *E. coli* HS1/2 and *E. coli* HS 3/104. We were looking for phages generating a stable fiber with specificity to the *E. coli* O87 type O-antigen that allows the phage to grow on *E. coli* HS3-104 host cells.

Plaques that produced good growth on HS3-104 but not on HS1/2 strain were identified. The phage mutant named DT571/2-ABF was purified by repeated plaque isolation and its phenotype was confirmed. The mutant phage produces normal plaques on *E. coli* HS3-104 with EOP close to 1.0 (in respect to plating on *E. coli* C600). The sequencing of the phage DT571/2-ABF *ltf* locus identified a large deletion from 71916 to 74004 nucleotide positions that fused the a.a. residues 1–663 of LtfA to the residues 288–767 of LtfB to form a single polypeptide chain (Figure 4). This result is fully compatible with our conclusion on the specificity of LtfA and LtfB receptor recognition domains. 

An extra copy of repetitive 19 a.a. motif (VDSALADKADIATTYTKME) was present within LtfA—deriving part of the fused LtfA-B protein. It resulted in three (and one more incomplete) copies of this motif instead of two and one incomplete copy in the wild type DT571/2 LtfA. It has to be noted that the stretch of these tandemly repeated a.a. motifs is located at 478–542 a.a. positions, immediately after the predicted region of homology to phage phiEco32 gp 14 protein that was suggested to form a contact with LtfB in the wild type phage (see above). We thus speculate that this duplication has a compensatory effect that fixed the negative effect of the LtfB inactivation on the fused Ltf protein assembly and/or stability. Interestingly, in phage DT5730(1) (see above) the quite similar deletion event was not accompanied by this duplication and led to the formation of a severely functionally compromised fiber with only a negligible activity.

## 4. Discussion

Summarizing all the data, we conclude that LtfA protein is essential for the phage growth on OPS-producing hosts but is not required on O-antigen deficient host strains. The data indicate that the LtfA receptors bind the O22-like OPS of *E. coli* 4s (in DT57C phage) or O87 type OPS of *E. coli* HS1/2. The LTF-OPS interaction is required for successful penetration of the phage central fiber with Hrs protein through the OPS layer to its BtuB receptor on the outer membrane surface. This conclusion is consistent with previously published data on phage T5 [25,26], but in contrast to T5, which can grow on OPS producing *E. coli* F strain even without LTFs [26], our isolates are strictly dependent on LTFs function to infect their OPS producing hosts. This indicates that the efficiency of the outer membrane surface protection by these O-antigen types is much higher than that of polymannose OPS studied by Heller and Braun [25,26] (see also [28]). The requirement of LtfA protein for *E. coli* HS3-104 (O81) infection is independent of the receptor recognition activity of this protein, and its role in this case appears to be limited to providing an attachment site for the LtfB assembly.

The apparent instability of LtfA protein in the absence of LtfB precluded us from directly demonstrating LtfB attachment *via* proteomic or morphological approaches, but the branched LTF organization in our phages can be reliably inferred from the existing body of bioinformatics and genetic evidence. This model is also in a good agreement with the observed complex morphology of the wild-type phage LTFs (the morphology of DT571/2 and DT57C was identical; Figure 5) that appears more complex than “conventional” phage T5 L-shaped fibers [17]. However, higher resolution images or reconstructions are required for the direct visualization of the LtfA and LtfB constituents of the phage lateral fiber. As far as we know, such a tail fiber arrangement has not been previously reported in Siphoviridae. Branched tail spike adhesins were characterized in *E. coli* K1 specific podoviruses [34] and in Vi-I-like myoviruses [35,36], and are believed to expand the viruses’ host ranges by containing receptor-recognizing proteins that act alternatively on different hosts. We also observed branched adhesins’ organization in bacteriophage G7C [29]. In this phage, gp 63.1 interacts with gp 66 and the latter is attached to the virion. The action of both proteins is required for the phage irreversible adsorption (our unpublished observations; manuscript in preparation).

As mentioned above, about half of the sequenced T5-related phage shares the LTF locus organization similar to DT57C or DT571/2 suggesting that an acquisition of branched adhesins is of significant ecological importance for this group of viruses. This speculation is particularly supported by the observation of the very frequent loss of the LTFs when the phage grows on the permissive strain and by the observed genetic instability of the double-gene *ltf* loci that leads apparently to a frequent spontaneous transition to a single-gene organization with the conservation of the function of one of the receptor recognition centres of the original branched fiber. The prevalence in nature of the fiber-containing T5-like phages, many of which have branched LTFs, is indicative of a strong stabilizing selection for these structures.

The fiber adhesins of bacteriophages have long been known as genetically plastic structures that can easily change in the course of virus evolution [37,38]. Bacteriophage fibers have also been shown to be the primary sites of mutation accumulation in laboratory phage-host co-evolution experiments [15] indicating that mutations in these proteins can have a strong impact on virus fitness. At the same time, in many siphoviruses like T5 [26] or lambda [39], the side tail fibers have been shown to be non-essential for infectivity under laboratory conditions. Moreover, the deletion of tail fibers may increase phage fitness in terms of rates of diffusion within diffusion-limiting media such as agar gel [39] resulting in faster spread of the phage progeny from the first lysed cells or may be otherwise restricted by the effective adsorption to the nearest host cells present in the medium.

This contradiction may be explained by the fact that, in natural ecosystems where the host density is much lower, the increased adsorption rates may provide to the phage a significant selective advantage, thus facilitating rapid evolution of the fibers that are often responsible for the reversible attachment stage of adsorption. In agreement with this assumption, receptor recognition by the LTFs is believed to be less specific than the ultimate infection-triggering interaction of the central fiber (or its associated adhesin) with the corresponding receptor. In fact, bacteriophage T5 LTFs recognize polymannose chains, thus mediating a quite rapid but non-specific attachment to the host cells producing this OPS [26].

In horse hindgut ecosystems, the strain-level diversity of the *E. coli* population is extremely high. In the subjects used for isolation of our series of T5-like phages, there were estimated to be as many as 700–1000 genetically distinct *E. coli* lineages simultaneously present in samples of faeces [14,40]. The fraction of the total coliform population that may serve as a host for any given bacteriophage may be limited to 2% or less ([14] and our unpublished results). Under these conditions, the density of the available hosts may be quite low (10^3^–10^4^ CFU per 1 mL of the gut contents) and the increased adsorption rate may strongly facilitate phage population stability in the gut preventing the rate of phage production from falling below the washout rate.

In contrast to our expectations, the LTFs of our T5-like phage isolates appeared to be involved in host range determination on an all or nothing basis. The data suggest that the OPSs of *E. coli* O22, O87 and probably O81 types provide the cells with a highly effective shield, completely protecting them from even very high phage exposure. In the case of the O22 glucosylated OPS of *E. coli* 4s strain, the treatment of cells by deacetylase enzyme derived from the bacteriophage G7C (Nikolay S. Prokhorov. Andrey V. Letarov., unpublished data) yields cells that are sensitive to DT571/2 phage, which otherwise is completely unable to infect these intact cells. Switching off the OPS O-acetylation in *E. coli* 4s by inactivation of *wclK* gene by IS element also makes the cell sensitive to all our T5-like phages and their *ltf* mutants ([28] and this work). These results demonstrate that the OPS shield efficacy can be modulated by a modification of the OPS such as O-acetylation. It has long been known that a change in OPS O-acetylation status due to phase variations [41,42,43] or lysogenic conversion [44,45,46] alters the phage sensitivity of enterobacteria. This effect, however, was explained only by the influence of the acetylation status on the specific interactions of phage receptor recognition proteins with the OPS receptors, while, to our knowledge, the possible impact on the efficiency of the non-specific protection of the other cell surface structures has never been considered in the literature.

Efficient penetration of phages to their final receptor through the O-antigen layer both in *E. coli* 4s and in *E. coli* HS1/2 requires a functional LtfA protein while LtfB is essential for adsorption on *E. coli* HS3-104 cells. The effect of purified LPS on adsorption of DT57C and DT571/2 bacteriophages, however, was only moderate, indicating that the interaction of the LTF with OPS is not very strong. The exact mechanism by which LTF generates a path through the OPS protective shield of the cell remains to be elucidated. The low (if any) inhibition of the phage adsorption by purified LPSs observed in our experiments is surprising and contrasts with published data [25]. Low affinity to the primary receptor may indicate that the penetration of the phage to its secondary receptor upon initial interaction with the cell is a quite rapid process and that long retention of the virion at the host cell surface mediated only by the LTFs is not required for infection. Identification of the OPS binding domain structure at least one of LtfA or LtfB proteins may provide an insight into the specific mechanisms involved in the LTF–O-antigen interaction.

As it was demonstrated by Lenski and Levin [47,48], *E. coli* B may become resistant to T5 without incurring any fitness cost under the laboratory conditions due to mutations in FhuA protein that serves as a secondary receptor for phage T5 [48]. The secondary receptor BtuB mutants were also selected in our experiments under the strong pressure of DT571/2 infection in the O-antigen deficient strain. Nevertheless, in natural environments such as the horse intestinal system, the non-specific resistance based on receptor shielding by cell surface structures including OPSs appear to be predominant over the mutational alterations of the BtuB receptor in bacterial avoidance of T5-like phage infection. This difference may be explained by the fact that in the horse gut the diversity of coliphages may be quite large ([14] and our unpublished data) and the impact of the T5-like phage infection cannot be separated from the impacts of other viruses. Instead of modification of FhuA or BtuB which could be the most beneficial response to T5-like phage infection alone in the *in vitro* experiments, it is more beneficial for the bacteria *in vivo* to respond to more complex challenges through the modification of external polysaccharide protective structures, which in turn drives the evolution of the T5-like bacteriophages LTFs.

From this point of view, the demonstrated LTF-independent infection by our phages of the mutant of *E. coli* 4s that produces a non-O-acetylated version of the O22-like O-antigen may be considered to be an ecologically important effect. This interference of different cell surface protection mechanisms may create an opportunity to design a highly effective therapeutical phage mixture using a narrow-spectrum phage recognizing the O-antigen in combination with other phages that target the resistant mutants possessing altered O-antigen structures. The advantage of such a scheme may stem from the fact that phages which are strictly dependent on highly represented host cell-surface molecules, such as OPS, frequently have high adsorption rates that would increase their ability to control the host population *in situ*. For example, phage G7C has a high adsorption constant on *E. coli* 4s of 2 × 10^−8^ mL·min^−1^ [29]. At the same time, the disabling of O-antigen modifications completely blocks specific adsorption of this phage and has apparently no significant fitness cost for the host. The presence of the second virus that would control this kind of mutated population(s) will force bacteria to acquire other mutations that are frequently associated with a significant decrease of phage fitness and virulence [49].

## 5. Conclusions 

The *in silico* and experimental analysis of double LTF genes function in DT57C and DT571/2 bacteriophages revealed that these genes, present also in the genomes of many other T5-like viruses, define the structure of branched lateral tail fiber carrying different receptor recognition domains on its LtfA and LtfB proteins. These domains recognize alternative primary receptors, expanding the host range of the virus. In bacteriophages DT57C and DT571/2 the LtfA proteins recognize (respectively) O-antigens of O22 or O87 types, and LtfB receptor molecule has been attributed as O81 type O-antigen. Switching off the synthesis of the O-antigen protective shield or its weakening by the alleviation of O-units O-acetylation (in *E. coli* 4s) overcome the absolute requirement for LTF presence for phage multiplication, allowing direct infection *via* the recognition of the secondary phage receptor —*E. coli* outer membrane protein BtuB, common for both of our phages.

## References

[1] Summers W.C., Kutter E., Sulakvelidze A. (2004). Bacteriophage research: Early history. Bacteriophages: Biology and Applications.

[2] Wang J., Jiang Y., Vincent M., Sun Y., Yu H., Wang J., Bao Q., Kong H., Hu S. (2005). Complete genome sequence of bacteriophage T5. Virology.

[3] Rabsch W., Ma L., Wiley G., Najar F.Z., Kaserer W., Schuerch D.W., Klebba J.E., Roe B.A., Laverde Gomez J.A., Schallmey M. (2007). FepA- and TonB-dependent bacteriophage H8: Receptor binding and genomic sequence. J. Bacteriol..

[4] Hong S.H., Wang X., Wood T.K. (2010). Controlling biofilm formation, prophage excision and cell death by rewiring global regulator H-NS of *Escherichia coli*. Microb. Biotechnol..

[5] Kim M., Ryu S. (2011). Characterization of a T5-like coliphage, SPC35, and differential development of resistance to SPC35 in *Salmonella* enterica serovar typhimurium and *Escherichia coli*. Appl. Environ. Microbiol..

[6] Raya R.R., Oot R.A., Moore-Maley B., Wieland S., Callaway T.R., Kutter E.M., Brabban A.D. (2011). Naturally resident and exogenously applied T4-like and T5-like bacteriophages can reduce *Escherichia coli* O157:H7 levels in sheep guts. Bacteriophage.

[7] Lee D.H., Lee J.H., Shin H., Ji S., Roh E., Jung K., Ryu S., Choi J., Heu S. (2012). Complete genome sequence of *Pectobacterium carotovorum subsp*. carotovorum bacteriophage My1. J. Virol..

[8] Effantin G., Boulanger P., Neumann E., Letellier L., Conway J.F. (2006). Bacteriophage T5 structure reveals similarities with HK97 and T4 suggesting evolutionary relationships. J. Mol. Biol..

[9] Sayers J., Calendar R. (2006). Bacteriophage T5. The Bacteriophages.

[10] Waller A.S., Yamada T., Kristensen D.M., Kultima J.R., Sunagawa S., Koonin E.V., Bork P. (2014). Classification and quantification of bacteriophage taxa in human gut metagenomes. ISME J..

[11] Niu Y.D., Stanford K., Kropinski A.M., Ackermann H.W., Johnson R.P., She Y.M., Ahmed R., Villegas A., McAllister T.A. (2012). Genomic, proteomic and physiological characterization of a T5-like bacteriophage for control of Shiga toxin-producing *Escherichia coli* O157:H7. PLoS ONE.

[12] Letarov A., Kulikov E. (2009). The bacteriophages in human- and animal body-associated microbial communities. J. Appl. Microbiol..

[13] Letarov A., Hyman P., Abedon S.T. (2012). Bacteriophages as a part of the human microbiome. Bacteriophages in Health and Disease.

[14] Golomidova A., Kulikov E., Isaeva A., Manykin A., Letarov A. (2007). The diversity of coliphages and coliforms in horse feces reveals a complex pattern of ecological interactions. Appl. Environ. Microbiol..

[15] Scanlan P.D., Hall A.R., Lopez-Pascua L.D., Buckling A. (2011). Genetic basis of infectivity evolution in a bacteriophage. Mol. Ecol..

[16] Koskella B., Brockhurst M.A. (2014). Bacteria-phage coevolution as a driver of ecological and evolutionary processes in microbial communities. FEMS Microbiol. Rev..

[17] Zivanovic Y., Confalonieri F., Ponchon L., Lurz R., Chami M., Flayhan A., Renouard M., Huet A., Decottignies P., Davidson A.R. (2014). Insights into bacteriophage T5 structure from analysis of its morphogenesis genes and protein components. J. Virol..

[18] Breyton C., Flayhan A., Gabel F., Lethier M., Durand G., Boulanger P., Chami M., Ebel C. (2013). Assessing the conformational changes of pb5, the receptor-binding protein of phage T5, upon binding to its *Escherichia coli* receptor FhuA. J. Biol. Chem..

[19] Flayhan A., Vellieux F.M., Lurz R., Maury O., Contreras-Martel C., Girard E., Boulanger P., Breyton C. (2014). Crystal structure of pb9, the distal tail protein of bacteriophage T5: A conserved structural motif among all siphophages. J. Virol..

[20] Bohm J., Lambert O., Frangakis A.S., Letellier L., Baumeister W., Rigaud J.L. (2001). FhuA-mediated phage genome transfer into liposomes: A cryo-electron tomography study. Curr. Biol.: CB.

[21] Braun V., Killmann H., Herrmann C. (1994). Inactivation of FhuA at the cell surface of *Escherichia coli* K-12 by a phage T5 lipoprotein at the periplasmic face of the outer membrane. J. Bacteriol..

[22] Decker K., Krauel V., Meesmann A., Heller K.J. (1994). Lytic conversion of *Escherichia coli* by bacteriophage T5: Blocking of the FhuA receptor protein by a lipoprotein expressed early during infection. Mol. Microbiol..

[23] Mondigler M., Ayoub A.T., Heller K.J. (2006). The DNA region of phage BF23 encoding receptor binding protein and receptor blocking lipoprotein lacks homology to the corresponding region of closely related phage T5. J. Basic Microbiol..

[24] Bradbeer C., Woodrow M.L., Khalifah L.I. (1976). Transport of vitamin B12 in *Escherichia coli*: Common receptor system for vitamin B12 and bacteriophage BF23 on the outer membrane of the cell envelope. J. Bacteriol..

[25] Heller K., Braun V. (1979). Accelerated adsorption of bacteriophage T5 to *Escherichia coli* F, resulting from reversible tail fiber-lipopolysaccharide binding. J. Bacteriol..

[26] Heller K., Braun V. (1982). Polymannose O-antigens of *Escherichia coli*, the binding sites for the reversible adsorption of bacteriophage T5+ via the L-shaped tail fibers. J. Virol..

[27] Golomidova A.K., Kulikov E.E., Prokhorov N.S., Guerrero-Ferreira R.C., Ksenzenko V.N., Tarasyan K.K., Letarov A.V. (2015). Complete genome sequences of T5-related *Escherichia coli* bacteriophages DT57C and DT571/2 isolated from horse feces. Arch. Virol..

[28] Knirel Y.A., Prokhorov N.S., Shashkov A.S., Ovchinnikova O.G., Zdorovenko E.L., Liu B., Kostryukova E.S., Larin A.K., Golomidova A.K., Letarov A.V. (2015). Variations in O-antigen biosynthesis and O-acetylation associated with altered phage sensitivity in *Escherichia coli* 4s. J. Bacteriol..

[29] Kulikov E., Kropinski A.M., Golomidova A., Lingohr E., Govorun V., Serebryakova M., Prokhorov N., Letarova M., Manykin A., Strotskaya A. (2012). Isolation and characterization of a novel indigenous intestinal N4-related coliphage vB_EcoP_G7C. Virology.

[30] Zdorovenko E.L., Golomidova A.K., Prokhorov N.S., Shashkov A.S., Wang L., Letarov A.V., Knirel Y.A. (2015). Structure of the O-polysaccharide of *Escherichia coli* O87. Carbohydr. Res..

[31] Flayhan A., Wien F., Paternostre M., Boulanger P., Breyton C. (2012). New insights into pb5, the receptor binding protein of bacteriophage T5, and its interaction with its *Escherichia coli* receptor FhuA. Biochimie.

[32] Mondigler M., Holz T., Heller K.J. (1996). Identification of the receptor-binding regions of pb5 proteins of bacteriophages T5 and BF23. Virology.

[33] Muhlenhoff M., Stummeyer K., Grove M., Sauerborn M., Gerardy-Schahn R. (2003). Proteolytic processing and oligomerization of bacteriophage-derived endosialidases. J. Biol. Chem..

[34] Leiman P.G., Battisti A.J., Bowman V.D., Stummeyer K., Muhlenhoff M., Gerardy-Schahn R., Scholl D., Molineux I.J. (2007). The structures of bacteriophages K1E and K1–5 explain processive degradation of polysaccharide capsules and evolution of new host specificities. J. Mol. Biol..

[35] Adriaenssens E.M., Ackermann H.W., Anany H., Blasdel B., Connerton I.F., Goulding D., Griffiths M.W., Hooton S.P., Kutter E.M., Kropinski A.M. (2012). A suggested new bacteriophage genus: “Viunalikevirus”. Arch. Virol..

[36] Kutter E.M., Skutt-Kakaria K., Blasdel B., El-Shibiny A., Castano A., Bryan D., Kropinski A.M., Villegas A., Ackermann H.W., Toribio A.L. (2011). Characterization of a ViI-like phage specific to *Escherichia coli* O157:H7. Virol. J..

[37] Sandmeier H., Iida S., Arber W. (1992). DNA inversion regions Min of plasmid p15B and Cin of bacteriophage P1: Evolution of bacteriophage tail fiber genes. J. Bacteriol..

[38] Trojet S.N., Caumont-Sarcos A., Perrody E., Comeau A.M., Krisch H.M. (2011). The gp38 adhesins of the T4 superfamily: A complex modular determinant of the phage’s host specificity. Genome Biol. Evol..

[39] Gallet R., Shao Y., Wang I.N. (2009). High adsorption rate is detrimental to bacteriophage fitness in a biofilm-like environment. BMC Evol. Biol..

[40] Isaeva A.S., Kulikov E.E., Tarasyan K.K., Letarov A.V. (2010). A novel high-resolving method for genomic PCR-fingerprinting of enterobacteria. Acta Nat..

[41] Broadbent S.E., Davies M.R., van der Woude M.W. (2010). Phase variation controls expression of *Salmonella lipopolysaccharide* modification genes by a DNA methylation-dependent mechanism. Mol. Microbiol..

[42] Wang L., Wang Q., Reeves P.R. (2010). The variation of O antigens in gram-negative bacteria. Sub-Cell. Biochem..

[43] Lerouge I., Vanderleyden J. (2002). O-antigen structural variation: Mechanisms and possible roles in animal/plant-microbe interactions. FEMS Microbiol. Rev..

[44] Hong J., Kim K.P., Heu S., Lee S.J., Adhya S., Ryu S. (2008). Identification of host receptor and receptor-binding module of a newly sequenced T5-like phage EPS7. FEMS Microbiol. Lett..

[45] Jakhetia R., Talukder K.A., Verma N.K. (2013). Isolation, characterization and comparative genomics of bacteriophage SfIV: A novel serotype converting phage from *Shigella flexneri*. BMC Genom..

[46] Sun Q., Knirel Y.A., Wang J., Luo X., Senchenkova S.N., Lan R., Shashkov A.S., Xu J. (2014). Serotype-converting bacteriophage SfII encodes an acyltransferase protein that mediates 6-O-acetylation of GlcNAc in Shigella flexneri O-antigens, conferring on the host a novel O-antigen epitope. J. Bacteriol..

[47] Lenski R.E., Levin B.R. (1985). Constrains on the evolution of bacteria and virulent phage: A model, some experiments and prediction for natural communities. Am. Nat..

[48] Bohannan B.J., Lenski R.E. (2000). Linking genetic change to community evolution: Insights from studies of bacteria and bacteriophage. Ecol. Lett..

[49] Leon M., Bastias R. (2015). Virulence reduction in bacteriophage resistant bacteria. Front. Microbiol..

